# From Generative Models to Generative Passages: A Computational Approach to (Neuro) Phenomenology

**DOI:** 10.1007/s13164-021-00604-y

**Published:** 2022-03-18

**Authors:** Maxwell J. D. Ramstead, Anil K. Seth, Casper Hesp, Lars Sandved-Smith, Jonas Mago, Michael Lifshitz, Giuseppe Pagnoni, Ryan Smith, Guillaume Dumas, Antoine Lutz, Karl Friston, Axel Constant

**Affiliations:** 1 Wellcome Centre for Human Neuroimaging, University College London, London, UK; 2 VERSES Research Lab and Spatial Web Foundation, Los Angeles, California USA; 3 School of Engineering and Informatics, University of Sussex, Brighton, BN1 9QJ UK; 4 Canadian Institute for Advanced Research (CIFAR), Program on Brain, Mind, and Consciousness, Toronto, Ontario, M5G 1M1 Canada; 5 Department of Psychology, University of Amsterdam, Science Park 904, 1098 XH Amsterdam, Netherlands; 6 Amsterdam Brain and Cognition Centre, University of Amsterdam, Science Park 904, 1098 XH Amsterdam, Netherlands; 7 Institute for Advanced Study, University of Amsterdam, Oude Turfmarkt 147, 1012 GC Amsterdam, Netherlands; 8 Lyon Neuroscience Research Centre, INSERM U1028, CNRS UMR5292, Lyon 1 University, Lyon, France; 9 Integrated Program in Neuroscience, Department of Neuroscience, McGill University, Montreal, Canada; 10 Division of Social and Transcultural Psychiatry, McGill University, Montreal, Canada; 11 Lady Davis Institute for Medical Research, Montreal Jewish General Hospital, Montreal, Canada; 12 Department of Biomedical, Metabolic and Neural Sciences, University of Modena and Reggio Emilia, Modena, Italy; 13 Center for Neuroscience and Neurotechnology, University of Modena and Reggio Emilia, Modena, Italy; 14 Laureate Institute for Brain Research, Tulsa, Oklahoma USA; 15 CHU Sainte-Justine Research Center, Department of Psychiatry, University of Montreal, Montreal, Canada; 16 Mila – Quebec Artificial Intelligence Institute, University of Montreal, Montreal, Canada; 17 Charles Perkins Centre, The University of Sydney, Sydney, Australia

## Abstract

This paper presents a version of neurophenomenology based on generative modelling techniques developed in computational neuroscience and biology. Our approach can be described as *computational phenomenology* because it applies methods originally developed in computational modelling to provide a formal model of the descriptions of lived experience in the phenomenological tradition of philosophy (e.g., the work of Edmund Husserl, Maurice Merleau-Ponty, etc.). The first section presents a brief review of the overall project to naturalize phenomenology. The second section presents and evaluates philosophical objections to that project and situates our version of computational phenomenology with respect to these projects. The third section reviews the generative modelling framework. The final section presents our approach in detail. We conclude by discussing how our approach differs from previous attempts to use generative modelling to help understand consciousness. In summary, we describe a version of computational phenomenology which uses generative modelling to construct a computational model of the inferential or interpretive processes that best explain this or that kind of lived experience.

## Introduction

In this paper, we pursue the naturalization of phenomenology using modelling techniques developed in computational neuroscience and biology. Specifically, we propose a new version of *neurophenomenology* as ‘generative passages’ (Lutz 2002; Roy et al. 1999; Varela 1997), based on methodological innovations that have emerged recently under the rubric of *generative modelling* (Friston 2019; Hesp et al. 2021; Sandved-Smith et al. 2021). Our approach represents a distinctive formulation of *computational phenomenology* because it applies methods from computational modelling directly to phenomenology. We present a specific application of computational phenomenology to what phenomenological philosophers have called ‘constitution’, see Sokolowski (1964). More precisely, we propose a model that maps phenomenological constitution onto the notion of inference, as it is used in generative modelling to infer the (causal) structure of the process generating some data or, more simply, making sense of data.

The structure of this paper is as follows. In the next two sections, we review the overall project to naturalize phenomenology. We then review the generative modelling framework and examine the broader meta-Bayesian approach to science and meta-science that follows from this approach. Next, we elaborate a distinctive perspective on neurophenomenology based on the broader meta-Bayesian perspective afforded by generative modelling. Generative modelling allows us to construct a formal, computational model of the interpretive process that must be in play for the type of experience that is disclosed to consciousness as such. More specifically, we propose to model phenomenological constitution as a form of (Bayesian or variational) inference. We seek to lay new groundwork for future research at the intersection of generative modelling and phenomenological inquiry.

## Naturalizing phenomenology

### A potted history

To situate the present proposal within the broader context of this research program, we offer a short, potted history of this project. Phenomenology is the descriptive study of conscious experience, its structure, its flow, and its dynamics (Husserl 1973; Dastur 1995; Moran 2005; Gallagher and Zahavi 2020). In its common non-technical usage, e.g., as it is used in physics and in biological psychiatry, the word ‘phenomenology’ refers to how things seem to some subject who is observing a phenomenon of interest. In this sense, the phenomenology of some event refers to that which presents itself to an observer, e.g., the presentation of low mood in a clinical context.

In the technical sense with which we are concerned here, the word ‘phenomenology’ has a narrower meaning. It refers both to a general methodological framework for studying experience and to a specific scholarly movement originating in Germany in the early twentieth century. Phenomenology is, in the words of the founder of the movement, Edmund Husserl, an attempt to describe conscious experience, without making any presuppositions about its nature (Depraz 1999). Without reducing phenomenology to any one specific method or claim, a cluster of things can be noted. Phenomenology is characterized by a specific conception of consciousness, as having something to do fundamentally with intentionality, that is, being directed-towards. In slogan form: all consciousness is somehow consciousness of something. In some of his writings, Husserl describes phenomenology as a systematic and rigorous attempt to describe the *essence* of various kinds of conscious experience or phenomena; where the essence of a phenomenon or thing comprises all of the essential or necessary properties that make that individual thing the kind of thing that it is (Husserl 2012b). Phenomenologists often describe their field of inquiry as the study of ‘lived’ experience, in the sense that it describes conscious experience as it occurs to an experiencer in the stream or flow of their existence.

Since the 1990s, there has been a systematic attempt to bring together the empirical sciences, and especially neurosciences, with phenomenology (Varela and Shear 1999; Schmicking and Gallagher 2009). For the most part, this project has taken the form of attempts to *naturalize phenomenology* (Petitot 1999; Roy et al. 1999; Zahavi 2013; Ramstead 2015; Gallagher 2012). Broadly speaking, to naturalize a domain of study or theory is to make that domain or theory coherent with the theories, methods, and practices in the empirical sciences, ultimately grounded in a physicalist worldview that assumes causal closure (i.e., only physical entities and forces exist and only those entities and forces can have effects on each other).

More specifically, naturalization can be understood in ontological, epistemological, or methodological senses (Ramstead 2015). The ontological naturalization of some theory or framework means that one reformulates all the properties, entities, and processes postulated by that theory in terms of those posited in the ontology of the empirical sciences; e.g., reducing them or (if one is non-reductionist) associating them systematically in some way to (e.g.) physical or chemical properties, entities, and processes. It is worth noting that ontological naturalization does not necessarily entail reductionism (although it is certainly compatible with it). In this context, reductionism (Ayala and Dobzhansky 1974; Kim 1989) is the position according to which the factors of causal relevance to some process are only the basic physical things and properties, from which other things emerge or upon which they supervene. One might, e.g., adopt an emergentist position with regards to physical entities (Bishop 2008; Juarrero 1999).

The epistemological naturalization of a theory or framework entails reformulation (Ramstead 2015). An epistemologically naturalized theory borrows its epistemological premises and assumptions about causal relationships between entities from the empirical sciences: on this view, bona fide knowledge of the natural world is ultimately grounded in causal explanations that appeal only to interactions between concrete physical entities (and, perhaps, appealing to mathematical entities; see Smith 2014b and Ramstead 2015, for discussion). In this context, an epistemologically naturalized phenomenology would borrow its core principles and forms of explanation from the natural sciences.

The methodological naturalization of a theory or framework is the attempt to bring it into methodological continuity with the empirical sciences in the study of some target domain; in this instance, it entails adopting methods, e.g., from psychology and neuroscience, to study the phenomenological aspects of first-person experience, which can constrain our modelling efforts. For the relations between these types of naturalism, see Ramstead (2015). The version of computational phenomenology developed here is a form of methodological naturalism. It is agnostic with respect to ontological and epistemological naturalism. More precisely, it takes advantage of methods developed in the natural sciences (i.e., generative modelling) to study phenomenological constitution.

### Varieties of naturalized phenomenology

The naturalization of phenomenology is a research program that aims to foster insightful cross-fertilization between disciplined first-person descriptions of conscious experience in phenomenological and hermeneutic philosophy and the scientific methods and tools of the natural sciences, in particular, of cognitive and computational neuroscience (Gallagher 2012; Petitot 1999; Ramstead 2015; Zahavi 2013). This project has been ongoing since the 1990s, with efforts to address its aims accelerating as consciousness increasingly regained its status as a prominent topic of study in the cognitive and brain sciences.

There have been three primary forms taken by the project to naturalize phenomenology—for a review, see Ramstead (2015). First, when working in neurosciences or in psychology, one might *draw from* phenomenology. ‘Front-loaded’ and ‘back-loaded’ phenomenology do just this (Gallagher 2003, 2010): these approaches leverage concepts and constructs that have been rigorously worked out at a descriptive, phenomenological level of analysis, which one can obtain from the work of classical phenomenologists, such as Husserl and Merleau-Ponty (Husserl 1991; Merleau-Ponty 1982), or from empirical methods such as ethnographic observation (Katz and Csordas 2003) or phenomenological interviewing (Petitmengin 2006), to inform quantitative research in cognitive neuroscience. ‘Front-loaded’ phenomenology applies constructs that are derived from phenomenological research to the third-person research practice. Thus, one front-loads insights derived from phenomenological analysis into one’s scientific protocol, to inform how experiments are designed; for instance, designing experiments to explore the neural correlates of the phenomenological distinction between sense of agency and sense of ownership of bodily experience (Gallagher 2003). In contrast, ‘back-loaded’ phenomenology uses previous results of experimental work in cognitive neuroscience as the basis for phenomenological analysis and thus re-interprets the results within the phenomenological conceptual framework (Braddock 2001).

The second primary form of naturalized phenomenology attempts to use new developments in mathematics to directly formalize the essential structures of phenomenological experience, especially those novelties that were not available when phenomenology first emerged as an intellectual movement, such as projective geometry, dynamical systems theory, information theory, computer science, etc. (Roy et al. 1999). This is known as formal phenomenology. Here, one only draws on phenomenology for its descriptions, which act as a target for naturalization. This approach thus begins with the rigorous descriptions of first-person, phenomenological experience. From these, one attempts to mathematically formalize their structure and dynamics—and, when possible, the processes that underlie them. To mathematically formalize the structure of phenomenological experience here means to identify formal mathematical structures that are isomorphic to, and according to proponents of this approach thereby explain, the structure of lived experience. Previous contributions to this project that explicitly draw upon phenomenology include the formalization of the geometry of perspective in visual consciousness via projective geometry and dynamical systems theory (Petitot 2004; Petitot and Smith 1996; Williford et al. 2018). A flagship example of this kind of thinking, albeit one that does not make explicit its relation to phenomenological analysis, is integrated information theory (Tononi et al. 2016). This theory begins from a number of phenomenological ‘axioms’ from which it derives claims about necessary and sufficient mechanisms for conscious experience (see Bayne 2018, for a critique). Other examples include the formalization of the perceptual property of ‘objecthood’ or ‘presence’ in terms of conditional or counterfactual predictions about sensorimotor contingencies (Seth, 2014), and the elucidation of the conscious experience of the present moment of time in terms of the computational processes that would have to be in play for a system to represent a temporally ‘thick’ state of affairs (Smith 2014a, b; Roy et al. 1999; Wiese 2017; Grush 2006).

Finally, the most well-known approach to the naturalization of phenomenology bears the name ‘neurophenomenology’. Neurophenomenology is an interdisciplinary approach to the study of conscious experience that integrates formal tools from mathematics, experimental tools from natural sciences, and first-person descriptions. This research program is characterized by taking the phenomenological, descriptive method of investigation seriously and applying it to empirical research in the natural sciences (Varela 1997). Indeed, the main feature of this approach is that it uses rigorous, phenomenological descriptions (Depraz et al. 2003) to generate first-person phenomenological or qualitative data amenable to neurocognitive methodology (Lutz 2002; Lutz et al. 2002; Gould et al. 2014).

Of particular relevance for our purposes here, the neurophenomenological approach emphasizes the pragmatic requirement of having recourse to specific methodologies to tackle well-known methodological difficulties associated with first-person reports—such as response biases and the influence of demand characteristics; see for example, Orne (1962). The methodologies that are leveraged by neurophenomenology include various first-person methods (e.g., mindfulness meditation, Varela 1996) and second-person methods to guide the collection of qualitative phenomenological data (Petitmengin 2006). The aim of this methodological approach is to create mutual, bidirectional constraints between approaches, with phenomenological methods providing constraints for what counts as an appropriate neuroscientific investigation of conscious experience, and reciprocally, with the discoveries of neuroscience orienting and informing phenomenological inquiry and descriptions. These systematic links have been called ‘generative passages’ (Lutz 2002; Varela 1997). The ultimate aim of neurophenomenology as the search for generative passages is to move beyond mere isomorphisms between first- and third-person accounts, and towards bona fide mutual epistemic accountability—and virtuous circularity—in the generation of scientific explanations.

In the words of the pioneer of neurophenomenology, Francisco Varela: “A more demanding approach will require that the isomorphic idea is taken one step forward to provide the passage where the mutual constraints not only share logical and epistemic accountability, but they are further required to be operationally generative, that is, where there is a mutual circulation and illumination between these domains proper to the entire phenomenal domain. This is to say, we must be prepared to be in a position to generate (in a principled manner) reduction analysis and eidetic descriptions that are rooted in an explicit manner to biological emergence” (Varela 1997). Mathematical tools provide an overarching, ‘ontologically neutral’ framework to systematically link and constrain the descriptions of phenomenology and the associated mechanistic dynamical processes (Lutz 2002; Varela 1997). The aim of neurophenomenology, then, is to move from a reductionist investigation into the neural substrates of consciousness towards an account of ‘generative passages’ that link the phenomenological level of description to the natural scientific one. Our project is to revisit the program of neurophenomenology as the search for generative passages in light of recent developments in computational modelling (i.e., generative modelling, described below).

### Naturalizing phenomenology: A non sequitur?

To a phenomenologically trained philosopher or the historian of ideas, the attempt to naturalize phenomenology might seem a bit strange, if not outright self-contradictory (Zahavi 2013; Ramstead 2015), because phenomenological philosophy started as an anti-naturalist research program (Kusch 1995). Indeed, the European phenomenological tradition as we know it started as a rejection of the claim that the empirical sciences could teach us anything at all about the essence of consciousness—and, for that matter, about any normative affair, e.g., the essence of knowledge (Husserl 1973).^1^

The short history of the matter is that the founder of phenomenological philosophy, Husserl, initially had worked very closely with Viennese descriptive psychologists such as Franz Brentano, whose aim was to study empirically the various sorts of conscious experience (Fisette 2007, 2015).^2^ Husserl was then introduced to the work of Kant, and after that intellectual encounter, largely adopted his aprioristic view on knowledge, becoming especially fascinated by the idea of synthetic a priori knowledge (i.e., a kind of knowledge that is true of a domain of experience, *independently* of any empirical knowledge of that domain). One salient example used by Husserl is that any experience of a sound, necessarily by virtue of its being a sound, will have the properties of timbre, pitch, and intensity. This is an essential or necessary truth pertaining to the experience of any and all sound whatsoever, the truth of which does not depend on whether one is verifying this fact in one’s own lived experience (or, as a special case, confirming this fact with the methods of the natural sciences); all experience of sound will necessarily conform to it, by virtue of what it *means* or *is* to be the experience of a sound (Husserl 2012b, 1973). This realm of ‘material a priori’ truths about conscious experience (Blouin 2021; Romano 2010) is, for Husserl, utterly irreducible to that of empirical truths, which are always subject to revision; and indeed, the latter kind of truth depends on the former, since the enterprise of acquiring knowledge of empirical things presupposes that they appear *first* in conscious experience (Romano 2010; Ramstead 2015). This is the philosophical core of Husserl’s transcendental idealism.

Husserl’s phenomenological project was precisely situated in an antagonistic relation to the notion that the natural sciences, or for that matter any empirical science, can make claims about the nature of knowledge or consciousness (Romano 2010). Those claims are subject to a kind of normativity, i.e., to normative standards about what it means to be true, to count as evidence, etc., that, according to Husserl, are always, and always must be for essential reasons, presupposed by the natural sciences (Husserl 1973; Ramstead 2015). Since the natural sciences depend on such normative standards, they cannot possibly be their source, lest we find ourselves in a vicious circle of reasoning (Husserl 2002). Husserl strongly rejected epistemological naturalism precisely for this reason: There exists, on his account, a domain of truths pertaining to the essential properties of things (consciousness, knowledge, and so on) that escapes, grounds, and overflows the domain of empirical knowledge (Ramstead 2015; Romano 2010).

The primacy of phenomenology with respect to the natural sciences was not only epistemological for Husserl, but also metaphysical (Blouin 2021). Husserl was interested in what he called the *constitution* of any object whatsoever, that is, (the structure and genesis of) the disclosure to conscious experience of any kind of thing, whether it be perceived, recollected, imagined, etc. (Sokolowski 1964). This problem is preliminary, on his account, to the empirical investigation of the contingent properties of things present within conscious experience, including, crucially, the brain and associated psychological processes. Husserl thus also rejected ontological naturalism and naturalization projects. Neurophenomenology would, therefore, have most likely been understood by Husserl’s followers as standing very much in opposition to Husserl’s own research program (Ramstead 2015; Zahavi 2013).^3^

How does this history square with naturalization projects? The project to naturalize phenomenology has proceeded mainly on the assumption that Husserl’s anti-naturalism resulted from historical circumstances (Roy et al. 1999). For example, on this account, Husserl’s claim that mathematical constructs could not formalize the fluid, changing morphology of phenomena as they are disclosed to consciousness (Husserl 2012b) would have been based on the paucity of mathematical tools available in his day to describe such forms, and can be readily revisited given recent advances allowing for such formal description, e.g., projective and fractal geometry, dynamical systems theory, and so on. For instance, one might appeal to dynamical systems theory and related modelling frameworks to model the dynamics of the ‘now-moment’ that is described in Husserl’s analysis of the constitution of inner time consciousness, with its retentional (past-oriented) and protentional (future-oriented) aspects; namely, the fact that our experience of the ‘now’ seems to spill out into the immediate past and future (Grush 2006). Such modelling techniques were simply not available to Husserl and his followers.

According to proponents of the naturalization project, then, it would be legitimate to reject Husserl’s philosophical project (i.e., transcendental idealism and the associated rejection of any attempt to explain the features of consciousness by appealing to empirical methods) and only to retain the rigorous descriptions of lived experience that he has left us in his voluminous work. Proponents of ontological naturalism, after all, reject the special (i.e., nonphysical) metaphysical status of the region of pure lived experience. Proponents of epistemological naturalism reject theories and frameworks that are not grounded ultimately in physical properties and would reject Husserl’s transcendental idealism, and accompanying theory of essences, for similar reasons. Methodological naturalists might simply not view the metaphysical and epistemological commitments of phenomenology as standing in the way of using methods from the natural sciences to study consciousness (but also, need not adhere to a more robust form of naturalism). Naturalist thinkers often appeal to the fact that later phenomenologists attempted to bridge the divide with empirical sciences as evidence for the contingent and merely historical aspect of Husserl’s position. This more permissive posture with respect to the contributions of the natural sciences to the study of conscious experience is most present in the work of later phenomenologists, such as Merleau-Ponty (Merleau-Ponty 1982). Despite not himself being a naturalist, and indeed, despite his commitment to Husserl’s transcendental idealism, the latter has had an immense influence on the recent course of cognitive neuroscience; e.g., in the development of embodied and enactive approaches to cognition, and in the development of neurophenomenology itself (Varela et al. 2016; Thompson and Varela 2001; Thompson 2010).

We will spell out a way of naturalizing phenomenology that extends the project of neurophenomenology as the search for generative passages, but that does not attempt (at least not in the first instance) to make claims about the neural structures and processes that underwrite, instantiate, enable the emergence of, or otherwise realize conscious experience.^4^ The version of *computational phenomenology* that we propose here is not aimed at the question of the ‘neural substrate’ of conscious experience—or at least, not as a starting point (although we do consider this important relation in Sect. 5 of this paper). It instead focuses on the structure and dynamic unfolding of lived experience per se, as it is described in phenomenological analyses. It does not merely draw on phenomenology as a source of qualitative data or as a source of concepts and constructs. Instead, it proposes computational models that *articulate* knowledge about lived experience originally drawn from rigorous approaches to gathering phenomenological data, such as Husserlian transcendental phenomenology, Heideggerian analysis of human existence or *Dasein*, Merleau-Ponty’s phenomenology of nature, or empirical questionnaire and interview methods from psychology and anthropology, among others. To do this, we write down a *generative model* of that phenomenology. We turn to generative modelling next.

## Generative modelling

On one view, generative models underwrite nearly all the physical and life sciences and, indeed, the scientific process itself. This is in the sense that all hypothesis testing, data assimilation, evidence accumulation, and more generally sense-making of observable outcomes rests upon the evidence for some model that generates data from latent or hidden causes. One sees generative models in statistics; ranging from simple general linear models used for classical inference through to high-end machine learning schemes (e.g., generative adversarial networks) (Roweis and Ghahramani 1999). The notion of a generative model unites many apparently disparate approaches to data and has been posited in one form or another as the basis of perception and understanding (D. M. MacKay 1956; Neisser 2014; Gregory 1980; Yuille and Kersten 2006).

Generative modelling (as opposed to discriminative modelling) is quintessentially probabilistic or Bayesian in nature—and usually comes in two flavors: *exact* versus *approximate*. Approximate Bayesian inference (a.k.a. variational Bayes) is the kind of model inversion realized in practice and can always be cast as extremizing a variational (free energy) bound on model evidence or marginal likelihood (Kass and Steffey 1989; Hinton and Zemel 1994; D. J. C. MacKay 1995; Beal 2003). Generative models are at the heart of things like the free energy principle and dynamic causal modelling (Friston et al. 2013; Friston 2019), in the sense that these approaches to ‘sense-making’ rest on model evidence: a function (i.e., marginal likelihood) of some data, under probabilistic beliefs about how those data were caused; i.e., a generative model. For example, dynamic causal modelling—originally developed in computational neuroscience (Friston 2019)—is gaining prominence in several other fields, such as cognitive and social psychology (e.g., Vasil et al. 2020) and epidemiology (e.g., Friston et al. 2020).

The general philosophy of generative modelling can be seen as deeply continuous with a broader shift in the way science is conducted, from correlation-based thinking to causal inference (Pearl 2018). Science was once concerned primarily with correlation, mostly under the influence of the philosophy of science of Hume (Hume 1777). However, of late, there has been significant interest, not to mention advances, in our ability to infer causal relations from data (Pearl 2018; Seth et al. 2015). Generative modelling speaks to this renewed call for a focus on causation. Indeed, generative models are just formulations of how causes generate consequences. The inversion of such models is known as (abductive) inference; namely, inferring the—usually unobservable—causes from—usually observable—consequences.

The idea of generative modelling is simple. This approach leverages Bayesian probabilistic models of the process that generated some data of interest, which we want to explain (the aptly named generative process). So, we want to model some data, which means that we want to find a configuration of latent causes of that data that best explains *causally* the variance in the data (Smith et al. 2020a, b, c). In the generative modelling scheme, this involves writing down a *probabilistic model* of the process by which some data of interest are generated. These are *generative models*, so-called because they are a way of representing the relations between some states that are of interest to us, which we believe have generated our data (Ashburner et al., 2003; Friston et al. 2017; Ramstead et al. 2019a). Generative models can be expressed in several equivalent ways, which have different uses depending on their application: we can define them in terms of (stochastic) equations of motion that define the dynamics or flows of states, or in terms of graphical probabilistic relations between states, as in Bayesian networks (Friston et al. 2017).

Technically, generative models harness our probabilistic beliefs about what might have caused our observations (i.e., prior beliefs) and our beliefs about the associations of such causes with specific observations, i.e., the conditional probability of those observations given those beliefs (which are called likelihoods). Priors comprise what we know about the base rates of occurrence of some phenomenon (e.g., in Montreal in general it rains 20% of the time). Likelihoods harness what we know about the probability of some data given some unobservable state of affairs (e.g., if it’s raining, then I’m likely to hear rain, feel wet, see raindrops, etc.). Priors and likelihoods together constitute our knowledge about the states that cause observations and how those states influence each other, and together compose the generative model (Friston et al. 2017).

**Fig. 1:**
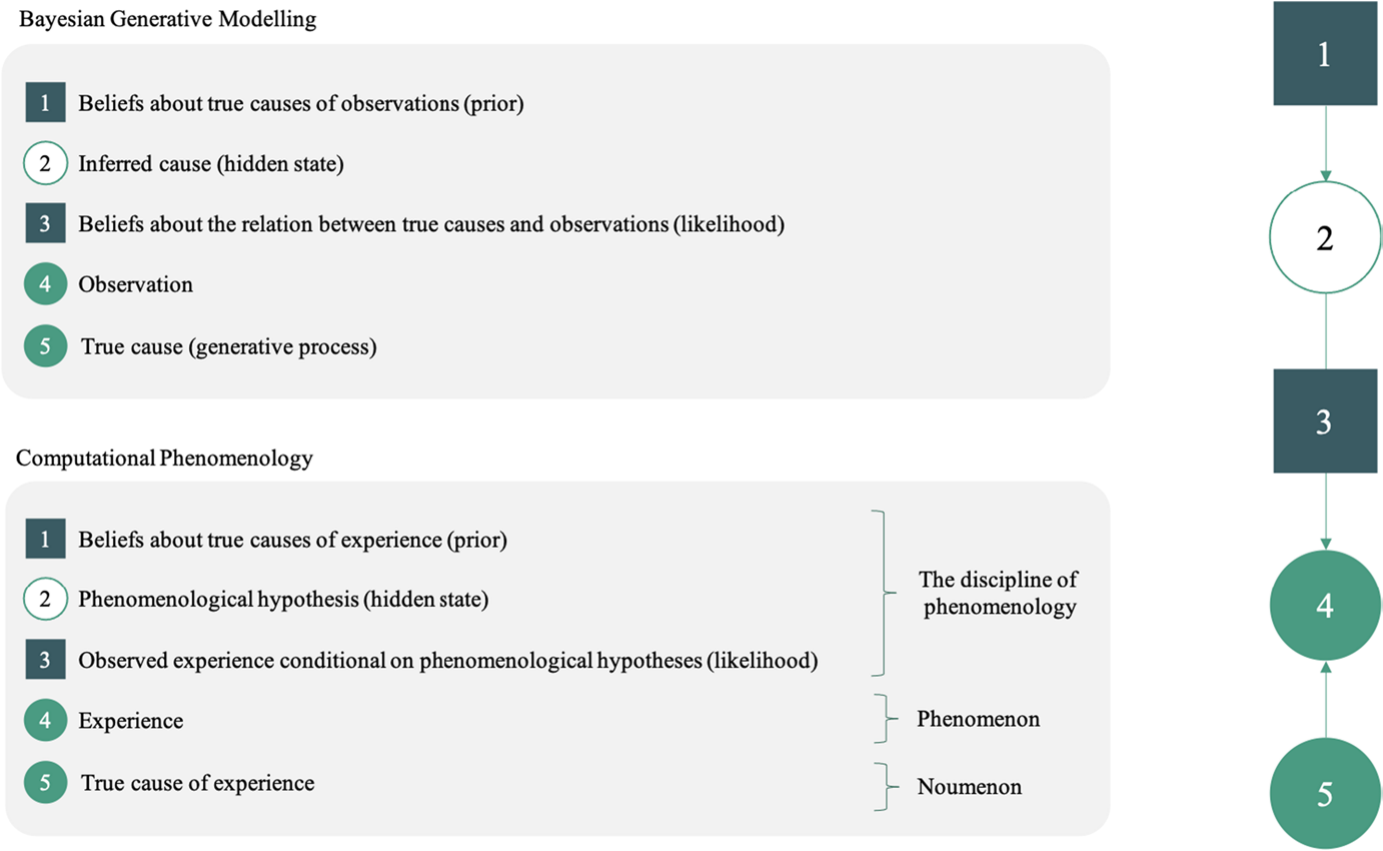
A generative model of phenomenological experience. According to Husserl, noema are constituted through a kind of interpretation process, where the ‘hyletic’ data of pure lived experience are ‘animated’ by a noetic intention. Computational phenomenology casts this process of disclosure as a kind of ‘inference’ process based on a generative model. **Right:** Basic components of generative modelling that are used in computational phenomenology, as they are typically deployed to model task behavior and neural processes (see in-text description). A generative model can be decomposed into priors and a likelihood, which together form (one decomposition of) a joint probability distribution over all states of a system. **Top left:** A generic generative model that is capable of perceptual inference. **Bottom left:** A simple generative model for computational phenomenology. Here, we have specified one prior, denoted **1**, which we could associate with phenomenological knowledge (e.g., claims of Husserlian, Merleau-Pontian, Heideggiarian, Bergsonian phenomenology about conscious lived experience and the structure of the lifeworld, i.e., what in the world that I inhabit typically causes this or that sensory data). The likelihood, which is denoted **3**, maps hyletic data onto that knowledge, in a conditional fashion (i.e., it specifies the kind of hyletic data that I would sense if this or that latent state was the cause of my experience). The hyletic data, then, corresponds in a straightforward way to the data or observable states in a generative model, denoted **4**. The noema or the phenomenological hypothesis that is mobilized to make sense of the hyletic data is labelled **2**. Through the dynamic flow of lived phenomenological experience, we form a belief about the cause of our lived experience in the world that we inhabit phenomenologically (which is, lest we forget, the world made up of noema; what Husserl called the lifeworld). In an objectivist (e.g., Kantian) metaphysics, the element denoted **4** is the ‘phenomenon’, generated by its true cause or ‘noumenon’, denoted **5**

Given some data, alternative models of the causal process are evaluated to assess how well each can explain the patterns in the data of interest. Technically, a score is computed for each iteration of the model, or each alternative parameterization of the model, which is called the model evidence or marginal likelihood. Practically, the (log) evidence for a model is usually scored with *variational free energy* (a.k.a., evidence lower bound). In sum, the model of the process that most plausibly caused our data is the one associated with the least free energy (or, equivalently, with the most evidence). Thus, we start from some data and infer the most probable model amongst those tested. See Fig. 1.

The generative modelling framework can be used to model phenomena at several different levels of description and can be applied to various types of data. These inference techniques were initially developed in Bayesian statistics and machine learning. The most probable model is identified by using an information theoretic metric—known as the evidence lower bound or (negative) variational free energy—that scores how well the model explains the variance in our data. When this framework is used to model continuous time-series data, such as neuroimaging time series, the associated inference technique is known as dynamical causal modelling (Ashburner et al. 2003). Generative modelling of this sort is a cornerstone of computational neuroscience (Stephan et al. 2010) and is being applied increasingly to domains outside the purview of neurobiology; e.g., to model the spread of the COVID-19 virus (e.g., Friston et al. 2020). When the data we want to explain are clinical diagnoses, this framework furnishes a computational nosology; and the associated model is a model of the diagnostic practice (Friston et al. 2017; Schwartenbeck and Friston 2016).

When we consider the sensory states of an (acting) organism as data to be explained, we are in the terrain of *active inference* (Friston 2019). In active inference, the generative model is a model of the organism acting in—or on—its environment, and its function is to infer which policy (i.e., plan or sequence of actions) the agent must be pursuing on the assumption that what it does must be minimizing the difference between anticipated outcomes—conditional on its course of action—and what it, a priori, it expects to sense (Ramstead et al. 2019a, 2020b; Tschantz et al. 2020). It turns out that this kind of active inference or sensing can also be understood as minimizing (expected) free energy; in the sense that actively soliciting the kind of data an agent-as-model can generate necessarily maximizes the evidence for that model—underwriting an elemental form of self-evidencing (Hohwy 2016). This formal framework is particularly attractive for computational phenomenology because it has impressive explanatory power and because it is also (at least arguably) compatible with an enactive and embodied view of the mind, which are central to phenomenology (Varela and Thompson 1991; Thompson and Varela 2001; Ramstead et al. 2020a). This completes our brief review of generative modelling.

## Computational phenomenology: Outline of a method to study phenomenological constitution

We now outline the main methodological steps that comprise our approach to constructing a formal phenomenology based on generative modelling. Essentially, our version of computational phenomenology is the form taken by generative modelling in cases where the *data or observations* that we want to explain by leveraging generative modelling is *our first-person phenomenology* itself. The idea that perceptual processes are closely related to conscious perceptual experience is often assumed implicitly in much of the existing generative modelling literature. Agents are said to be experiencing, for example, mental actions such as reading (Friston et al. 2017), their own emotional states (Hesp et al. 2021), or an episode of mind wandering (Sandved-Smith et al. 2021). In short, my first-person phenomenology is the best explanation for or interpretation of (i.e., hypothesis about) my engagement with the sensorium. In the same sense that perception can be cast as evaluating the sensory evidence for specific hypotheses about what is causing our sensations (Gregory 1980, 1968), those things that are experienced (what Husserl called *noema*) are the result of particular inferences or interpretations (*noesis*) about raw sensory data (*hyle*). The aim of the present work is to spell out this implicit assumption more explicitly. We unpack this presently.

In Husserl’s account (Husserl 2012b, 1989), each lived experience that is constituted in the flow of experience has two ‘sides’ to it. These are ‘subject-side’, comprising the *noesis* and the *hyle*, and the ‘object-side’, namely, the *noema* (Sokolowski 1964; Husserl 2012b). The subject-side of experience comprises everything that is indubitably and fully accessible, ‘in the flesh’, within any immediate ‘slice’ of conscious experience. The subject-side itself comprises, first, the ‘raw’, uninterpreted sensory data of experience—which Husserl called ‘hyletic’ data, from the Greek word for matter or substance, *hyle*, and which we might today call ‘qualia’—and second, the attitude or intention with which we aim at that sensory material, e.g., with doubt, with credence, etc., which Husserl called the *noesis*. The *noema* is the thing that is actually experienced over time, as it is constituted phenomenologically in the flux of lived experience. In Husserl’s framework, which is (at least according to some exegetical interpretations) reminiscent of Kant’s, noema are constituted thanks to the hyletic matter or raw sensory impression being ‘interpreted’ or ‘apprehended’ (*Deutung*/*Auffasung*) over time, through the intentional component of the noesis. Essentially, the intentional component of the noesis ‘threads together’ the raw sensory material into a temporally extended experience of a thing, the noema. Thus, the thing perceived (the noema) is constituted through the continuous interpretive activity of noesis, as a synthetic unity of various sensory profiles (*Abschattungen*) (Husserl 1991).

According to Husserl, our commonsense notion of an observer-independent world of scientific causes is actually *derived from*, and *secondary to*, the notion of a world of things that disclose themselves through their sensory appearances, i.e., the actual things that we encounter in our world of lived experience, or what Husserl called the ‘lifeworld’ (Husserl 1970). In this view, the crisis of foundations that shook the scientific world around the start of the twentieth century was a direct result of our collective forgetting of the fundamental status of the lifeworld with regards to the derived world of scientific, objective things.

What does this have to do with generative modelling? Here, we leverage generative modelling to formalize central aspects of the construct of *constitution* from Husserlian phenomenology (Sokolowski 1964). We propose that it is possible to write down a *generative model of the conscious, phenomenal experience* of any kind of thing whatsoever, extending the scope and methods of phenomenology via formalization and computational modelling.

In computational phenomenology as presented here, it is the *hyletic data* themselves, the raw qualia of lived experience, that are understood as the data needing to be explained by appealing to a model of their causes. Crucially, adopting the methods of generative modelling does not *ipso facto* commit one to an objectivist or naturalist ontology or epistemology. Indeed, in our approach, the causes of hyletic data are not assumed to be independently existing objects that do not disclose themselves directly to conscious experience (a Kantian noumenon). Rather, these causes are assumed to be the *noemata* themselves, not the things in the ‘objective’ world ‘behind’ the sensory appearances (see Fig. 1). In other words, on our account, the first-person phenomenology associated with some type of experience of things in the lifeworld is the result of a process of interpretation or inference from the raw sensory data to the noema that they disclose.

As discussed above, in typical generative modelling, when some data are explained by appealing to inference (i.e., inversion of generative model^5^), this explanation is cast as identifying the most probable, ‘hidden’ causes that could have generated the data to which we are privy. Usually, this involves reconstructing or inferring the causes ‘out there’, in the external world, of what we register through our sensory apparatus (again, see Fig. 1). In computational phenomenology as described here, this kind of constructivist explanation is associated instead directly with our phenomenological experience. In other words, the data that are being generated by the model in this case are the hyletic data themselves. The interpretation that we make of hyletic data then allows us to understand these data as the sensory profiles of a thing that discloses itself in conscious experience through such profiles.

Our basic claim is this: The disclosure or constitution of things in conscious experience thus can be modelled as an interpretive or inferential process, of subjects’ moving from the pure data of sense experience (or hyletic data, in Husserlian terms) to the interpreted things of lived experience (to noema, in Husserlian terms) (Husserl 2012b). Here, disclosure is an attribute of the process whereby phenomenological interpretations are reached on the basis of observed experience and prior knowledge about the lifeworld (i.e., on the basis of a generative model of the lifeworld).

To make explicit the structure of the disclosure of some type of lived experience, here, means finding the ‘phenomenological hypothesis’ or ‘interpretation’ or ‘noema’ that best makes sense of that pure lived experience. Heuristically, it is almost as if we were implicitly answering the question: “Among all the possible causes of my experience, and given the conditional relations that hold between my experience and my (often implicit) knowledge of what is generating it, as partly contingent on my embodiment; given all this, what is the interpretation (noema) for which I have the most evidence?” Of course, this question is not something literally asked by a subject or ego or self but stands in for a set of relationships that can be described without necessarily invoking an explicit self that is doing the asking. Under the proposed model, answering that heuristic question means, formally, performing the corresponding inference as described by the generative model.

To summarize so far: Computational phenomenology attempts to use generative modelling to formalize aspects of lived experience that are described in the writings of phenomenologists and others interested in the rigorous description of first-person experience. The core methodological tool here is the construction of a generative model of the kind of experience that is of interest; here, the phenomenological constitution of this or that type of lived experience. Starting from a rigorous description of some form of conscious lived experience, what we aim to do is to construct a generative model that can generate the structure and dynamics of the specific lived experience that we are interested in modelling (here, the disclosure of things to conscious experience). In the specific example case that we present to illustrate how one would apply the method, we suggest that we can use the generative modelling approach to propose a formal model of phenomenological constitution as described by Husserl (with the caveat that is but one interpretation of this notion of Husserl’s among many). See Fig. 1.

Consider a simple example, from Husserl’s extensive catalogue (Husserl 1991). What is it to hear some sounds as being the notes of a melody? On Husserl’s account, it is to interpret ‘raw’ auditory sensory material (labelled ‘4’ in Fig. 1) as disclosing a melody over the course of experience (labelled ‘2’ in Fig. 1). First, the *hyle* wells up in consciousness, as a sensation in my auditory field. In the flux of consciousness, any response involves an attitude with which that datum is interpreted (labelled ‘1’ and ‘3’, which make up the generative model, or joint probability of experience and our knowledge about its cause), here in the mode that Husserl called ‘doxastic’, i.e., I believe that what I am experiencing exists (i.e., the belief, or phenomenological hypothesis that my experience is caused by the sensory material). The noesis ‘animates’ the raw sensory material, and the result is that I experience a fully constituted melody, which I interpret as that thing that disclosed itself through my lived experience. This corresponds to the inversion of the generative model that gives us the hidden state, or phenomenological hypothesis. Computationally speaking, it is the temporal depth of the generative model (i.e., the fact that the model includes beliefs about the temporally deep structure of the generative process) under consideration that allows observed notes to be bound into a temporally extended melody. In other words, the evidence for my perceptual hypotheses lives both in the recent past and future; necessarily calling on notions of postdiction and prediction.

Crucially, this construction allows us to think about the *structure of intentionality*, which is a critical component of phenomenological philosophy. In Husserl’s terminology, the structure of lived experience is such that noemas are constituted through an *intentional* process of noetic apprehension of the raw ‘hyletic’ data; where intentional denotes the ‘towardness’ of consciousness, the fact that is always ‘about’. The prior beliefs that are part of what constitutes the generative model might be regarded as ‘point towards’ the ‘causes’ of lived experience in what Husserl called the lifeworld (Husserl 1970)—whatever the ontological status of these causes (physical things, abstract things, imaginary things, things believed, things hoped, things doubted, etc.) may be.

Our generative modelling approach to computational phenomenology also encourages consideration of the role action plays in shaping perceptual experience—when inference is extended to include action as a means of resolving prediction error (i.e., active inference). For example, Parr and colleagues argue that the temporal dynamics of perceptual experience in Troxler fading and binocular rivalry can be accounted for by active inference in the context of accumulating uncertainty (Parr et al. 2019). Accommodating the role of action is appealing from the perspective of philosophical phenomenology, since it brings into view the embodied and enactive aspects of perceptual experience highlighted by phenomenologists such as Merleau-Ponty (1982).

So far, our generative modelling approach to phenomenology dovetails mainly with the second of the primary forms of naturalized phenomenology, i.e., formal phenomenology, in that it attempts to use new developments in mathematical and computational modelling to directly formalize the essential structures of phenomenological experience. We now discuss how this approach could be extended to consider the brain, body, and world, allowing us to move towards a more demanding kind of naturalized phenomenology based on the establishment of mutual epistemic and scientific accountability and cross-fertilization between the first- and third-person perspectives.

## Towards an account of generative passages: Moving from phenomenology to neurobiology

The aim of this paper was to outline a conceptual and methodological framework for a particular approach to computational phenomenology. Our approach adopts a computational approach to the study of phenomenological experience, as it is understood and described by phenomenologists, and without making specific metaphysical assumptions about the metaphysics and mechanistic underpinnings of lived experience (i.e., assumptions about whether and how lived experience is related to the processes ongoing in the body, brain, and world). This pragmatic approach focuses on pursuing a scientific research agenda, namely, the formal description of the structures and flow of conscious experience, while acknowledging the complexity of the metaphysical and epistemological debates around the explanatory gap that is said to yawn between phenomenological and neuroscientific data.

Computational phenomenology as described here does not commit to ontological or epistemological naturalism, as it does not claim that the models developed under its auspices is ‘real’, ‘true’, or ‘natural’ in any sense. The generative models at stake in computational phenomenology are not necessarily models of the underlying neurophysiology that may realize lived experience, the so-called neural correlates of consciousness. However, they do provide constraints concerning the (minimally) necessary properties of systems that undergird a particular conscious experience of interest (Hohwy and Seth 2020). This is a subtle, but important distinction that may differentiate our approach from other forms of naturalized phenomenology. At the same time, having identified, modelled, and validated a generative model of a phenomenological feature of lived experience with reproducible first-person data could create novel, well-defined kind of mutual constraints between phenomenological and neuroscientific descriptive registers (what we have called ‘generative passages’), enabling us to explore and potentially identify some of its neurobiological underlying processes, as we now discuss.

Our account dovetails nicely with radical new approaches in metaphysics that aim to get beyond the explanatory gap by studying the way lifeworlds are constituted for the living creature itself, from its point of view (Bitbol 2021). Indeed, it could lead to giving a formal definition of precisely what it means for an organism to have a *point of view*—that is ‘its own’ (Metzinger 2003).

Importantly, however, the models of computational phenomenology also lay groundwork for linking phenomenological descriptions with the mechanisms of the body and brain—as a further step in the modelling effort. The resources of the broader Bayesian framework in which generative modelling is situated can indeed allow us to map the formal account of the interpretive process that gives meaning to our sensory experiences, gleaned from computational phenomenology, onto neurobiological activity. Of particular interest is the hierarchical integration of information and its Bayesian formulation, which are inherent to generative modelling (Friston 2019; Ashburner et al. 2003).

We suggest that this modelling heuristic can help to take steps towards relating systematically the interpretive processes described in the first instance by computational phenomenology and the inferential architectures that underwrite the structure and dynamics of the human brain and body. This is because of the overarching framework adopted in computational phenomenology—namely, generative modelling—can allow us to relate systematically the inferential architectures identified via this technique and the (minimal) suite of neural processes that must be in play for such inferences to take place.

In ordinary generative modelling, we identify candidate neural structures and processes that might realize the inferences at play in perception and action. These identifications can take place at various levels of description, from a precise differentiation of circuits, mechanisms, or regions which realize the distinct inferences described by the generative model, up to more abstract algorithmic descriptions which map more loosely onto neuroanatomy and neurophysiology (Suzuki et al. 2017). While the version of computational phenomenology developed here remains agnostic about the exact way by which neuronal dynamics map onto phenomenology, there is a clear relation between the more classical approach and the one being proposed here, in the form of mutual (model) constraints. The idea is that for any particular phenomenon, the underlying generative model of phenomenology will constrain the space of allowable generative models specifying a specific realization or process model (Andrews 2021).

Of note is that the present approach and target of explanation should be thought of as distinct from the target of explanation in other, closely related research on the neural correlates of consciousness (e.g., Mashour et al. 2020). The target of explanation in the latter literature instead pertains to the associations between neural representations of particular contents and reportable phenomenology, and why there are such associations in some cases but not others. For example, subcortical representations of fast timescale, moment-to-moment changes in blood pressure (linked to the baroreflex) do not appear to have associated conscious percepts, while representations of other objects/events (e.g., of a face or of one’s heartbeat) can be associated with phenomenological experience, but sometimes are not (e.g., due to brief presentation time or a lack of attention—despite still showing content-specific priming effects) (Park et al. 2014).

Active inference models have also recently been used to offer insights into these questions, via the construction of hierarchical generative models that can reproduce empirical results in this field through neural and behavioral simulations (Whyte and Smith 2020; Whyte et al. 2021). While this approach is successful at providing some accounts of ‘what happens *in* the black box’ during the presence or the absence of conscious percepts, its contributions remain limited for explaining ‘what happens *for* the black box’ i.e., *what it is like to be* a black boxed agent that is having this or that type of experience (Roy et al. 1999). These questions of lived experience and the ‘what-it-is-like’ are at the core of the present project of using generative modelling as an approach to directly model the structure and dynamics of phenomenological lived experience itself.

## A model of phenomenology, or a model experiencing itself modelling?

From this perspective, it is important to distinguish (at least) two interpretations of the generative models used in the version of computational phenomenology outlined here. On one interpretation, these models are taken to be useful scientific descriptions of the process of constitution from an external perspective (that of the experimenter). In this case, the model is a tool for the study of the phenomenological mind. A second interpretation is that the models are understood as descriptions of the actual inferential processes at play; here, the model is a description of the processes undergone by the phenomenological mind itself. This means that one can take these generative models (1) simply as scientific models, tools for conducting research, without any assumption that they reflect the metaphysics of consciousness and lived experience, or (2) implying some metaphysics of mind.

Crucial to note is that we computational phenomenology is not a metaphysical account of consciousness. We are not suggesting that phenomenological constitution *merely is* or is equivalent to a process of inference (or at least, not here). After all, as Natalie Depraz remarks (personal communication), the concept of phenomenological constitution in Husserl’s phenomenology is in a fundamental sense orthogonal to the explicit procedure of inference as it figures in scientific and philosophical reasoning. We suggest that interesting aspects of lived experience as described by phenomenology—and have exemplified the methodology by considering the the phenomenological (Husserlian) notion of constitution and how it can be modelled usefully as a form of inference. This is ultimately a methodological naturalist claim, not a metaphysical stance.

Now, it is tempting to ask whether a generative model can—in and of itself—be equipped with a computational model, such that inferences on this model might have their own phenomenological quality. The overall viewpoint is closely aligned with Safron’s (2020) ‘integrated world modelling theory’, which draws on generative modelling and integrated information theory to elucidate ‘what it is like’ to be an agent that is performing inference under a generative model. In other words, are ‘beliefs’, ‘experience’, and ‘true causes’ further hypotheses we use to explain our inferential dispositions?

Our approach is compatible with this but does not imply it. On one view, the math is not the territory (Andrews 2021). That is, one can view this modelling approach as the development of mere tools to study the mind. On another view, at least for certain kinds of perceptual inference, it must be the case that ‘beliefs’, ‘experience’, and ‘hidden causes’ are hypotheses that we actually use to explain our inferential dispositions; namely, those that rest upon *attention*. Almost universally, the inversion of generative models in neurosciences calls upon the selection of certain sensory data through a form of covert action (Rizzolatti et al. 1987; Parr and Friston 2019). However, this covert action must itself be inferred; mandating the realization of the hypothesis ‘I am attending this’. This is only tenable if ‘attention’ is a constituent of the underlying generative model. Could a similar argument be applied to states of mind like ‘I am experiencing this’, ‘I doubt this’—namely, the *neotic* attitudes or intentions with which we aim at sensory evidence?

## Limitations and future directions

This work was deliberately limited to the description of a very simple generative model that would offer formalism to explain phenomenological ‘constitution’ as inference under a generative model. This involved a prior that could correspond to (one interpretation of) claims of Husserlian phenomenology. However, one can imagine generative models that integrate claims from diverse traditions. The architecture of generative models allows one to model various aspects of the causes of data dynamics, for instance, by adding to the hierarchy of priors, or by modelling the unfolding over time of the data (i.e., temporal experience) in relation to a generative process (the process that generated our data, here our phenomenological experience) that can also be enriched with various kinds of priors. The priors that one can add to the architecture of the generative model or process may offer the possibility of mapping a variety of claims coming from different traditions. For instance, one might argue that the generative process (that generates the data to be explained, in our case hyletic data) might be the focus of a formalized Merleau-Pontian phenomenology, since the latter emphasizes the ‘invisible’ underbelly of a situated, worldbound consciousness and its roots in the material body, which together lend structure to conscious experience (Merleau-Ponty 1968); or might argue that Husserlian phenomenology’s emphasis on self-grasping in phenomenological self-experience lends itself well to hierarchical modelling (Husserl 1991, 2012a, b; Sandved-Smith et al. 2021; Hesp et al. 2021).

This latter example raises the exciting possibility of extending the present modelling approach to model the domain of phenomenological reduction, or épochè (Husserl, 2013), a central feature of the phenomenological enterprise. More technically, this domain of first-person investigation is grounded in the distinctions in experience between *transcendent* and *immanent* mode of appearance (Husserl 2012b). Husserl’s insight here is that some forms of experience are more corrigible or prone to error than others. For instance, I look at my red coffee mug. As I do, the coffee mug is constituted as an object through the adumbrations provided by the sensory (hyletic) material in my field of vision (and in my other sensory fields). I could be dreaming or hallucinating: the mug may not be real. However, even if my interpretation of the hyletic data as disclosing a mug is erroneous or mistaken, *while I am seeing this red expanse* in my visual field, it is utterly indubitable that I must be *seeing the color red*, this red expanse of my field. Hence, Husserl’s distinction: One perceives an object as transcendent when it appears to me as ‘real,’ existing in itself, ‘there’ in ‘objective’ space and time (for example as modelled by Seth (2014) in terms of counterfactual sensorimotor predictions). This transcendent mode of perception covers most of our daily dealings with things in our lifeworld and is described in contemporary psychology terms as reification (Lutz et al. 2015), absorption (Tellegen and Atkinson 1974), subjective realism (Lebois et al. 2015), or transparency (Metzinger 2003). Thus, computational phenomenology might even be used to model the self-reflective aspects of the performance of the épochè.

On the other hand, one can attempt to suspend one’s beliefs about what is being examined, to turn one’s attention to the *manner in which* this event manifests itself to me, as a *direct* and *immediate* phenomenon. This change of attitude or ‘conversion’ is known as the phenomenological reduction, or épochè, in the Husserlian framework, and it is the cornerstone of Husserl’s descriptive methodology (Husserl 2013, [b] 2012). This notion of immanence describes the intimate mode of access by which conscious activity can perceive itself as being spontaneously changing or being affected. The ensuing meta-perspective on mental states has been labelled, in contemporary terms, as phenomenological reduction (Varela 1996), decentering (Bernstein et al. 2015), cognitive defusion (Fletcher and Hayes 2005), mindful attention (Papies et al. 2012), dereification (Lutz et al. 2015), or opacification (Metzinger 2003). A formal model of meta-awareness and attentional control using hierarchical active inference has recently been proposed to model this shift between the transcendent and immanent mode of experience (Sandved-Smith et al. 2021). We believe this lays the groundwork for a bona fide scientific study of the phenomenological perspective or attitude itself.

A topic for further inquiry concerns the nestedness, specificity, and scalability of these mechanisms to account for specific phenomena (Ramstead et al. 2019a, b, 2020a, b). For instance, the phenomenon of a heard sound is likely best associated with neuronal architectures (e.g., pyramidal cell populations) situated close to the auditory cortex that encode a recognition density over the hidden states representing the likely causes of the sound, i.e., ‘the flow or ordered, intentional succession of these sensory materials discloses or constitutes a melody being played on the piano’ (Friston and Friston 2013). However, phenomenological access to aspects of the self that are related to longer timescales (e.g., the album-listening self, the narrative self) is more likely associated with activity in temporal lobes and central brain circuits, such as the default mode network (Carhart-Harris and Friston 2010).

Thus, prior to the direct mapping of generative models of phenomenology to their neurobiological counterparts, a more refined set of models considering the interplay of these generative models on different spatial and temporal scales is required. While this work has been done previously to some extent (Ramstead et al. 2019a, b, 2020a, b), it remains a topic of further inquiry to explore how the lived experience underwritten by these different generative models interact. After all, we are not only aware of sounds or of a narrative self but of a rich stream of a variety of such phenomena and their interactions, many of which are nested percepts (e.g., a book containing chapters that contain paragraphs, and so forth). Future work would need to consider deep-temporal percepts (as in Friston et al. 2017) and how these various streams coalesce to shape momentary subjective experiences. For example, generative models can capture the way in which internal ideation about potential future events affects one’s current affective states and, recursively, how these direct one’s ideation about the future (Hesp et al. 2020). This type of generative model provides various points of connection for empirical data, as it can be related to current affective states (e.g., through heart rate, biomarkers such as cortisol), imagined future scenarios (e.g., through fMRI), and behaviors (e.g., through eye gaze tracking, self-reports). Another related line would explore how generative models geared towards physiological regulation and control might explain the characteristic phenomenology of emotional and mood-related experiences (Seth and Tsakiris, 2018). Here, points of connection may again be drawn between features of control-oriented prediction and empirical biomarkers, such as heart rate (Smith et al. 2021).

Finally, while our illustrative example was restricted to an individual subject, generative models can also integrate social interaction (Schilbach et al. 2013; Smith and Lane 2020; Smith, et al. 2020a, b, c; Veissière et al. 2019) and thus allow us to approach the intersubjective dimension of lived experience (Constant et al. 2019). Such a formalization can ultimately help in the understanding of the subjective experience associated with clinical conditions, especially psychiatric conditions and their treatment (Smith et al. 2019a, b, c, d; Smith et al. 2019a, b, c, d; Smith et al. 2019a), where this intersubjective dimension is fundamental (Dumas et al., 2020; Smith et al. 2019a, b, c, d). Indeed, generative models such as the one we have described have even begun to be used in empirical studies to account for experiential differences in mental health (Smith et al. 2020; Smith et al. 2020a, b, c). Such extensions of our approach to the realm of emotions (e.g., Seth and Tsakiris 2018) and social interactions—both healthy and not—arguably expands its scope to include some of the richest phenomenological aspects of our experience.

As discussed, our approach can also be extended in less orthodox Husserlian directions. The interpretive or inferential structure described in this paper can be recursively folded in on itself, to construct hierarchical percepts in which, above some perceptual threshold, noemata that are stable enough at one spatiotemporal scale can serve as ‘raw’, uninterpreted hyletic data for the next. Think, for example, of the noema associated with a person’s body parts, which in turn provide hyletic data to inform the noema of that person as a whole. It is in this hierarchical scheme that our formulation can start to speak to the classical ‘binding problem’ of cognitive neuroscience (Revonsuo and Newman 1999; Bartels and Zeki 2006), both in terms of segregation of features between percepts and their combination into a single experience. Here, the consideration of recursive hierarchical formulations is essential for multi-level percepts such as, for example, ascertaining whether another agent is benevolent or not, which requires first delineating what parts of the world belong to them and are under their (potential) control. The conception of self that flows from this view coheres less with the unitary subject of transcendental consciousness in Husserl’s phenomenology, but follows more closely the spirit of Merleau-Ponty and other phenomenologists.

The proposed approach also may allow us to better understand and characterize the phenomenology of unusual mental states cultivated by particular cultural or religious practices, with specific generative models embedding traditional understandings about mental states and their causal factors (Veissière et al. 2019; Ramstead et al. 2016). For example, the application of this approach to the phenomenology of Buddhist contemplative practice is an interesting prospect given that there exists a rich body of knowledge about the structure of consciousness in the Abhidharma and Yogacara scriptures. Indeed, Varela himself considered the disciplined examination of conscious experience through contemplative practice as a major source of inspiration for his neurophenomenology program (Varela and Thompson 1991).

## Conclusion

We have proposed a new approach to formal phenomenology based on generative modelling, under the rubric of ‘computational phenomenology’. In our approach, generative models are not, in and of themselves, descriptions of the physical processes that realize or implement phenomenological descriptions. Rather, they describe the process of disclosure to consciousness of a given type of experience. We hope that the approach that we have outlined here can contribute to renewed mutual circulation between phenomenology and the sciences that study the mind.

Ours is not the first attempt to show that (e.g., a Husserlian) phenomenology could be cast in terms of computational modelling (e.g., Dreyfus and Hall 1982; Petitot 2004, 1994). Our approach to computational phenomenology relates to neurophenomenology by using the resources of the generative modelling framework to describe the interpretive or ‘inferential’ processes and dynamics that best fit the phenomenology of interest.

In our approach, generative models are not—without the use of auxiliary assumptions and appeal to other work—models of what the brain does when the phenomenology of interest is disclosed. At this level, our project is continuous with the formal phenomenology described in the second section. They provide heuristics or hypotheses for thinking about how the structure of experience arises and unfolds, and how thinking about the structure of experience is in turn constitutive of our experience (at least in part). When considered as the first step of a broader project aiming to establish generative passages between phenomenological and neural-embodied descriptions, the project dovetails with Varela’s approach to neurophenomenology.

## Footnotes

1 Readers should note that Husserl’s body of work is immense and spans several decades. In this paper, we are presenting one specific (and exegetically debatable) interpretation of Husserl’s phenomenological work. An anonymous reviewer notes that our interpretation of Husserl is in line with standard neo-Kantian ones. We do not mean to contribute to exegetical discussion about the works of Husserl in this paper. We wish to exemplify a version of computational phenomenology by using it to model aspects of lived experience that are captured in phenomenological descriptions; and Husserl’s descriptions serve as a means to illustrate our approach. More specifically, the interpretation of Husserl that we present here is based on neo-Kantian readings of his earlier works, especially his views on phenomenological constitution in the *Logical Investigations* (Husserl, 1913) and in the first volume of the *Ideas pertaining to a Pure Phenomenology and to a Phenomenological Philosophy* (Husserl, 2012a). We do not mean to endorse Husserl’s transcendental phenomenology or any particular interpretation of it, but rather to illustrate a specific version of computational phenomenology.

2 We are agnostic with respect to the relation between the objects of everyday, first-person conscious experience (what Husserl calls noema) and ‘real objects out there in the world’ that might be disclosed to consciousness in a perspectival manner, ‘through’ or ‘behind’ the noema. We are proposing to use descriptions of the early Husserl, and in particular Husserl’s early analysis of phenomenological constitution (Sokolowski, 1964), to illustrate our approach, without committing to a specific metaphysics of conscious experience. Husserl (2012a) tells us that those entities that are experienced consciously are noemata, which together make up the world that we inhabit from a first-person perspective—what he later calls the ‘lifeworld’ (Husserl, 1970). In our everyday lived experience, we interact with places, people, furniture, etc., none of which are exactly the same as the aggregates of atoms and molecules with which the natural sciences are concerned. For Husserl, our experience of the kinds of objects postulated in the natural sciences, such as atoms, is grounded in, and made possible by, our experience with the objects that populate the lifeworld of everyday experience (noemata). That is, the objects of natural science are themselves constituted on the basis of noema; they are merely further elaborations of the things already given to us 'in the flesh', scientific hypotheses pursued with a specific (i.e., naturalistic) attitude. Methodologically, this is consonant with the view espoused here. From the point of view of computational phenomenology as presented here, what matters is the structure of experience and what is experienced, not what might lie ‘behind’ or ‘underneath’ that experience. Thus, we do not need to make any assumptions about the ‘object itself’, as distinct from the associated noema.

3 One might cast our approach to phenomenology as a version of what Husserl called “phenomenological psychology” (Husserl, 2012b, 2013). Phenomenological psychology is the counterpart to transcendental phenomenology in the empirical sciences. According to Husserl, such a science is a coherent project because it is always possible to conduct eidetic descriptions of phenomenological experience without carrying out the épochè, in the natural attitude. That is, we remain within the natural (or naturalistic) attitude, but we carry out phenomenological descriptions to elucidate the essence or essential properties of lived experience. The difference between the two is that, unlike the descriptions of transcendental phenomenology, which are about the region of pure lived experience and the pure transcendental subject of those experiences, the descriptions of phenomenological psychology are instead about the concrete human being that is undergoing these experiences. In other words, transcendental phenomenology aims to identify the invariant universals of lived experience in general, while phenomenological psychology aims to describe the invariant structure of the experience of individual human beings. For Husserl, this opened the door to a scientific study of the essential (eidetic) properties of types of conscious experience, considered as happening to specific concrete egos. Our own project is continuous with phenomenological psychology, but also distinct from it. It is continuous because, in most cases, the descriptions of computational phenomenology are also intended to be descriptions of the experiences of concrete human beings; our approach is, after all, meant to add to the empirical sciences that study consciousness. However, it is distinct from phenomenological psychology. This is because phenomenological eidetic descriptions of lived experience provide a target for our computational modelling; so, our method is not purely descriptive in the Husserlian sense (i.e., entirely descriptive and eidetic), but it instead imports (and indeed, has as its core) elements from generative modelling.

4 Note that the aim of the paper is not to provide an exegesis of Husserl’s transcendental phenomenology. As indicated, Husserl’s phenomenological descriptions are meant to be illustrative, i.e., to serve as a placeholder for a wide variety of phenomenological descriptions from different traditions in phenomenology.

5 Model inversion is a technical way of describing inference. A generative model is sometimes referred to as a forward model that maps from causes to consequences. Model inversion maps from consequences to causes and entails inference; namely, updating prior beliefs about the (unobservable) latent causes of (observable) consequences to form posterior beliefs: a process sometimes referred to as Bayesian belief updating.
